# Changes in cerebral vascular reactivity following mild repetitive head injury in awake rats: modeling the human experience

**DOI:** 10.1007/s00221-024-06907-7

**Published:** 2024-08-20

**Authors:** Nicole Bens, Praveen Kulkarni, Craig F. Ferris

**Affiliations:** 1 Center for Translational Neuroimaging, Northeastern University, 360 Huntington Ave, Boston, MA 02115 USA; 2 Departments of Psychology and Pharmaceutical Sciences, Northeastern University, Boston, MA USA

**Keywords:** Concussion, Cerebral small vessel disease, Momentum exchange, Hypercapnia

## Abstract

The changes in brain function in response to mild head injury are usually subtle and go undetected. Physiological biomarkers would aid in the early diagnosis of mild head injury. In this study we used hypercapnia to follow changes in cerebral vascular reactivity after repetitive mild head injury. We hypothesized head injury would reduce vascular reactivity. Rats were maintained on a reverse light-dark cycle and head impacted daily at 24 h intervals over three days. All head impacts were delivered while rats were fully awake under red light illumination. There was no neuroradiological evidence of brain damage. After the 3rd impact rats were exposed to 5% CO_2_ and imaged for changes in BOLD signal. All imaging was done while rats were awake without the confound of anesthesia. The data were registered to a 3D MRI rat atlas with 171 segmented brain areas providing site specific information on vascular reactivity. The changes in vascular reactivity were not uniform across the brain. The prefrontal cortex, somatosensory cortex and basal ganglia showed the hypothesized decrease in vascular reactivity while the cerebellum, thalamus, brainstem, and olfactory system showed an increase in BOLD signal to hypercapnia.

**Supplementary Information:**

The online version contains supplementary material available at 10.1007/s00221-024-06907-7.

## Introduction

The Centers for Disease Control and Prevention report around 2.9 million people in the United States suffer from traumatic brain injury every year, with ca. 61,000 deaths reported in 2019 alone (Peterson et al. 2014). There is an expanding literature on the behavioral and neurobiological consequences of repetitive mild head injuries that incur while playing organized sports, car accidents, falls or in active military service. Indeed, these types of mild head impacts account for over 75% of all brain injuries (Cassidy et al. 2004). The guidelines from the Centers for Disease Control and Prevention, World Health Organization, and American Congress of Rehabilitation Medicine for diagnosing mild head injuries include self-reports of transient confusion, disorientation, impaired consciousness, or dysfunction in memory around the time of the injury with no neuroradiological evidence of structural damage in the brain (Cassidy et al. 2004; Silverberg et al. 2023). The effects of a single mild head impact are difficult to assess, and any neurobiological, cognitive or behavioral problems can resolve within hours of the injury. However, a more insidious problem arises when the brain is exposed to repeated mild head impacts. Repeated mild TBI (rmTBI) is associated with more severe and protracted cognitive, motor, and behavioral complications that can last months-to-years (Beaumont et al. 2012). Even after the remission of symptoms, there is accumulating evidence that persistent brain injuries (Vergara et al. 2017) carry increased risk of dementia, including Alzheimer’s disease, chronic traumatic encephalopathy, and Parkinson’s disease (Gardner and Yaffe 2015).

Fundamental to brain health is autoregulation of cerebral blood flow in the face of increases and decreases in systemic blood pressure. At the level of the neurovascular unit, homeostasis is maintained by local changes in vascular reactivity and capillary blood flow in response to the surrounding metabolic environment. A simple biomarker for evaluating the health of cerebral blood vessels is a hypercapnic challenge (Kassner and Roberts 2004). The smooth muscle in cerebral arteries is highly responsive to the levels of CO_2_ in the blood. Catalyzed by carbonic anhydrase, CO_2_ is combined with water to create carbonic acid which dissociates into bicarbonate and hydrogen ions. This acidic environment locally intensifies the vasodilatory effects of adenosine and vascular smooth muscle potassium ion conductance across cerebral blood vessels. This results in a passive expansion of blood vessels, decreased resistance, and heightened blood flow. The BOLD (Blood Oxygen Level Dependent) effect is influenced by this process. Importantly, when exposed to higher levels of CO_2_, there is no change in metabolic oxygen consumption. Consequently, the alteration in the MRI signal caused by the increased blood flow in the brain is directly linked to the change in the partial pressure of CO_2_. The change in BOLD signal in response to heightened CO_2_ levels is a straightforward and reliable technique for evaluating the cerebral vascular reactivity (CVR) in functional imaging studies (Davis et al. 1998).

The present study used 5% hypercapnic challenge and BOLD imaging in awake female rats to assess CVR following three mild head injuries, one each at 24 h intervals. To make these preclinical studies more relevant to the human experience repetitive mild head impacts, were performed on fully awake rats, during the circadian L-D cycle when they are normally active. There was no neuroradiological evidence of skull damage or brain contusion. We hypothesized these mild repetitive head impacts would reduce CVR; instead, the vascular sensitivity to CO_2_ challenge was heterogenous with some brain areas being more sensitive and others less.

## Methods

### Animals

Eighteen female Sprague Dawley rats (250–300 g) ca. 100 days of age were purchased from Charles River Laboratories (Wilmington, MA, USA), housed on a reverse 12:12 light-dark cycle (lights off at 9:00 h), maintained in ambient temperature (22–24 °C) and provided with food and water ad libitum. Rats were randomly assigned to two experimental groups of equal numbers: (1) sham controls with no head impacts, and (2) head impacted. All experiments were conducted under dim red illumination between 10:00 h and 18:00 h to avoid the transitions between the L-D dark cycles. Females were chosen over males because of the dearth of preclinical literature on head injuries using this sex. All animals were cared for in accordance with the NIH Guide to the Care and Use of Laboratory Animals. Methods and procedures used in this study were pre-approved by the Northeastern University Institutional Animal Care and Use Committee protocol # 21-0824R. The protocols used in this study complied with the regulations of the Institutional and adhere to the ARRIVE guidelines for reporting in vivo experiments in animal research (Kilkenny et al. 2010). Animals were monitored daily over the duration of the study for general health, food and water consumption. A 15% loss in body weight was set as a humane endpoint.

### Head impacts

Head impacts were generated with a pneumatic pressure drive, 50 g compactor to reliably produce the 7.4 m/s impact velocities for mild rat head injury. The kinetic energy at impact was ca. 1.37 joules. We have used this model to publish on the neuroradiological effects of repetitive mild head injury in rats (Kulkarni et al. 2019; Cai et al. 2021; Leaston et al. 2021). The impact piston was directed to the top of the skull, midline, in the approximate area of Bregma while rats are fully awake. This model is comparable to CHIMERA developed for mouse mild head injury (Namjoshi et al. 2017). The difference being these rats were hit while fully awake and during the active period of their circadian cycle. All rats showed normal ambulatory behavior within seconds of being placed into their home cage after head impact. There were no mortalities. Sham and head impacted rats were pretreated with slow-release buprenorphine (0.1 mg/kg IP) 30 min prior to head impact to minimize the pain over the three-day protocol. Rats were subjected to three mild head impacts separated by 24 h each as previously described (Kulkarni et al. 2019; Leaston et al. 2021). All rats were imaged within one hr after their 3rd head injury. Neuroradiological evidence of the edema on the skin of the skull at the point of impact is shown in Supplementary Fig. 1. There was no evidence of brain damage.

### Acclimation for awake imaging

To alleviate the stress related to head restraint, rats were familiarized with the restraining setup a week prior to their actual imaging session. The design of the restraining system featured a cushioned head support, eliminating the need for ear bars, which helped minimize the discomfort experienced by the animals and reduced motion artifacts. These familiarization sessions were conducted daily for five consecutive days. Rats were briefly anesthetized with 2–3% isoflurane while being secured in the head holder. Their forepaws were secured using surgical tape. Once fully conscious, the imaging system was placed inside a black opaque box, referred to as a “mock scanner,” for 30 min. During this time, a recording of the MRI pulse sequence was played to simulate the magnet bore and the imaging protocol. A significant decrease in respiration, heart rate, motor movements, and plasma corticosterone levels was observed when comparing the first and last acclimation sessions (King et al. 2005). This reduction in autonomic and somatic indicators of arousal and stress improves signal resolution and image quality. Because of motion artifact two rats from each group were eliminated. A time course of motion artifact is provided in Supplementary Fig. 2. All fourteen of the rats included in the study are combined to show the mean ± SE for the X, Y and Z axis for 150 image acquisitions. The imaging parameters set the in-plane resolution of a pixel at was 312 μm. The average displacement for the 14 rats ranged between 70 –27 μm. We eliminated subjects that had motion exceeding the dimension of one or two pixels. The SPM motion correction algorithm used in this study (see below) was used to adjust for the changes shown here.

### Image acquisition

Five-six rats were imaged in a day. Each day had a mix of sham and impacted animals known by all the investigators. Animals were scanned at 300 MHz using a quadrature transmit/ receive volume coil built into the rat head holder and restraining system for awake animal imaging (Ekam Imaging, Boston MA USA). A video of the rat preparation for imaging is available at www.youtube.com/watch?v=JQX1wgOV3K4. The design of the coil provided complete coverage of the brain from olfactory bulbs to brain stem. Radio frequency signals were sent and received with a quadrature volume coil built into the animal restrainer (Ekam Imaging, Boston MA, USA) (Ferris 2022). The design of the restraining system included a padded head support obviating the need for ear bars, helping to reduce discomfort while minimizing motion artifact. Imaging sessions were conducted using a Bruker Biospec 7.0 T/20-cm USR horizontal magnet (Bruker, Billerica, MA, USA) and a 2 T/m magnetic field gradient insert (ID = 12 cm) capable of a 120-µs rise time. At the beginning of each imaging session, a high-resolution anatomical data set was collected using the RARE pulse sequence [22 slice; 1.0 mm; field of view (FOV) 3.0 cm; matrix size 256 × 256; repetition time (TR) 2.5 s; echo time (TE) 12 ms; NEX 2; 3 min acquisition time]. Functional images were acquired using a multi-slice half-Fourier acquisition single-shot turbo spin echo (HASTE) pulse sequence. Bruker *Paravision* automatically finds the basic frequency, shims, power requirements for 90° and 180° pulses and sets the receiver gain. A single scanning session acquired 22 slices, 1.0 mm thick, every 6.0 s (TR), using an effective TE of 48 ms, FOV 3.0 cm, matrix size 96 × 96, NEX 1, and repeated 150 times for a total scanning time of 15 min. The in-plane pixel resolution was 312 µm^2^. Each scanning session was continuous, starting with five min baseline followed by five min of 5% CO_2_ exposure and five min following cessation of CO_2_.

### Motion correction

SPM motion correction was used with the following setting: Quality = 0.97; Spatial separation: 0.45 mm; Smoothing FWHM: 0.65 mm; Interpolation: 2nd degree B-spline; Reslice Interpolation: 4th degree B-spline; Images were registered to mean image.

### Data analysis

Data were co-registered to a mean functional image using SPM8’s co-registrational code with the following parameters: quality, 0.97; smoothing, 0.35 mm; and separation, 0.5 mm. Gaussian smoothing was performed with a FWHM of 0.8 mm. Preprocessed functional files were then exported to Medical Image Visualization and Analysis (MIVA) for registration and segmentation. Images were aligned and registered to a 3D rat brain atlas, which is segmented and labeled with 171 discrete anatomical regions. The alignment process was facilitated by an interactive graphic user interface. The registration process involved translation, rotation, and scaling independently and in all three dimensions. Matrices that transformed each subject’s anatomy were used to embed each slice within the atlas. All pixel locations of anatomy that were transformed were tagged with regions of interest in the atlas. This combination created a fully segmented representation of each subject within the atlas. The inverse transformation matrix [Ti]-1 for each subject (i) was also calculated. For voxel-based analysis, the percent change in BOLD signal for each independent voxel was averaged for all subjects with a baseline threshold of 1% BOLD change to account for normal fluctuation of BOLD signal in the rat brain under the awake condition (Brevard et al. 2003). A composite image of the whole brain representing the average of all subjects was constructed for each group for ROI analyses, allowing us to look at each ROI separately to determine the BOLD change and the number of activated voxels in each ROI. The *t*-test statistics used a 95% confidence level, two-tailed distributions, and heteroscedastic variance assumptions. As a result of the multiple *t*-test analyses performed, a false-positive detection controlling mechanism was introduced. This subsequent filter guaranteed that, on average, the false-positive detection rate was below our cutoff of 0.05. Statistical *t*-tests were performed on each voxel (ca. 15,000 in number) of each subject within their original coordinate system. The average signal intensity in each voxel of the first 5 min of baseline (1–50) was compared to 5–10 min (acquisitions 51–100) CO_2_ exposure. We refer to the number of voxels in each brain area that showed a significant increase in BOLD signal above threshold as volume of activation. The mean volume of activation is the average of all rats for each experimental condition for that brain area. Volume of activation was compared across brain regions e.g. cerebellum, prefrontal ctx, basal ganglia (see Supplementary Data File 1 for organization of brain regions).

### Data availability

All data generated or analyzed during this study are included in this published article and its supplementary information files.

## Results

### Vascular reactivity

**Fig. 1:**
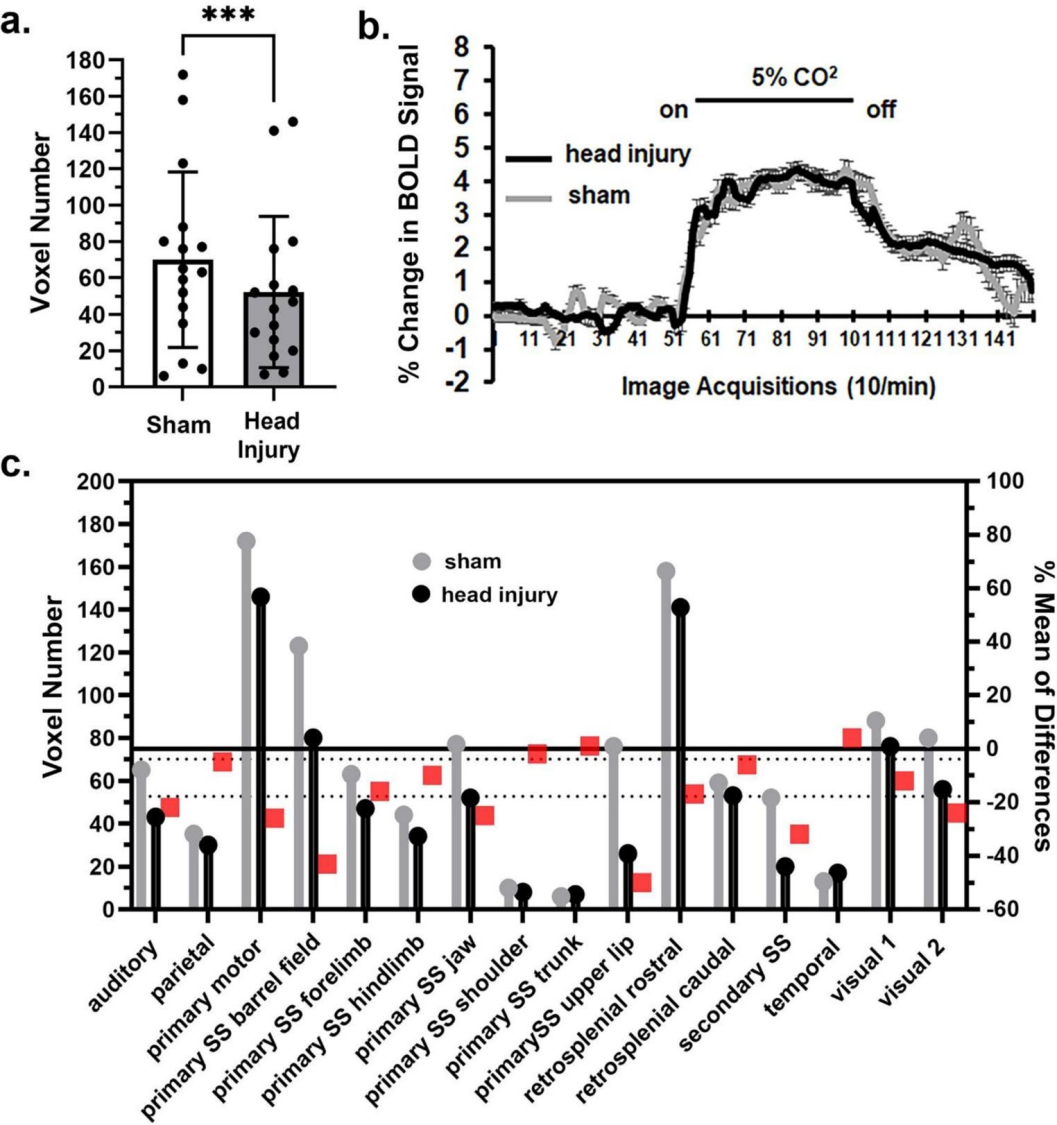
Prefrontal cortex response to CO_2_. Shown in 1a are bar graphs (mean ± SD) of the mean volume of activation i.e. number of voxels comprising the prefrontal cortex in sham and head injury rats in response to 5% CO_2_ challenge as shown in 1b. The mean is the average of 8 brain areas that comprise the prefrontal ctx. The difference in the mean volume of activation for each of these individual areas is shown in the 1c. The red boxes are the % mean of differences. The dashed horizontal lines are 95% confidence intervals. * *p* < 0.05

**Fig. 2:**
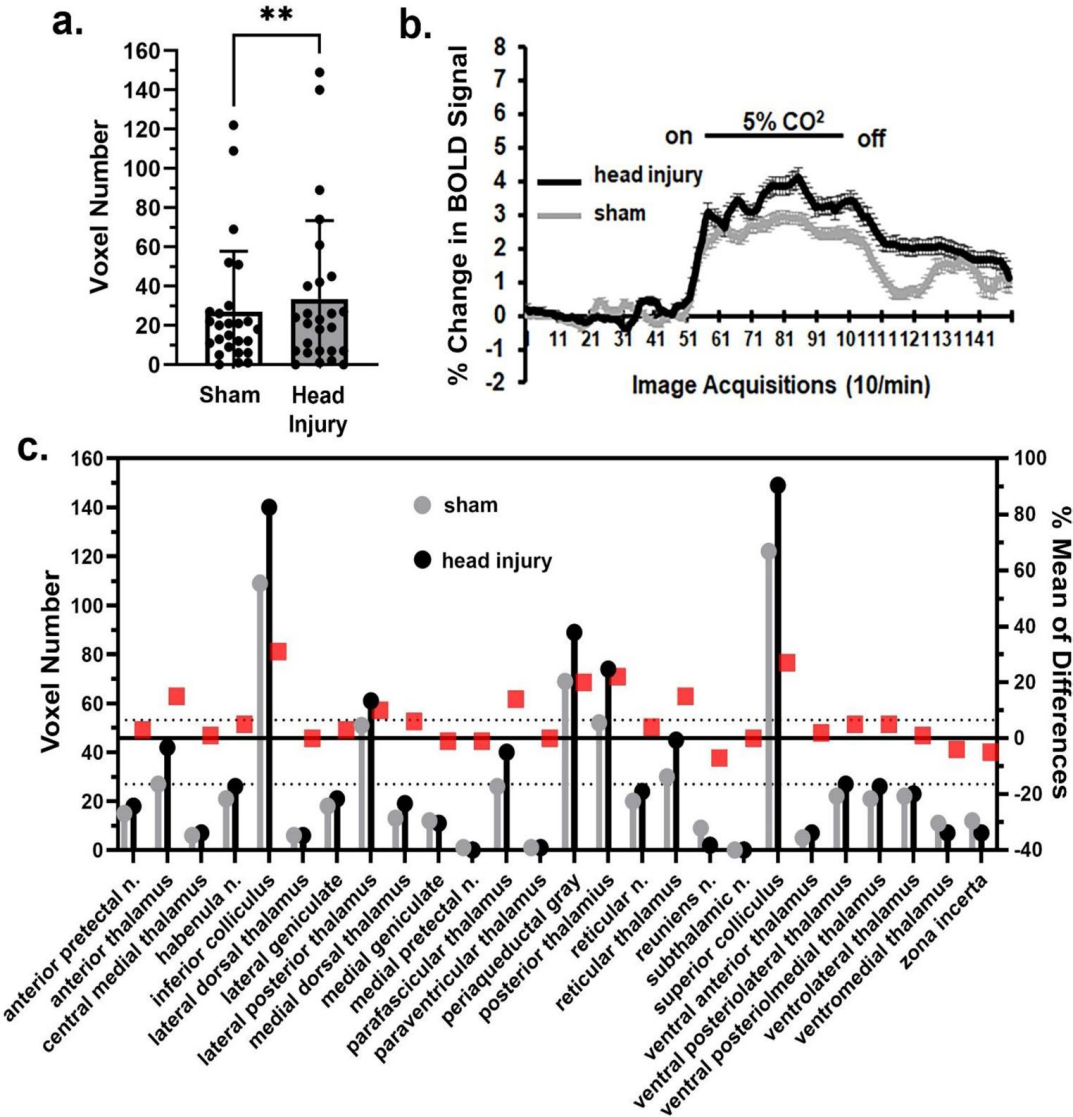
Sensorimotor cortex response to CO_2_. Shown in 2a. are bar graphs (mean ± SD) of the mean volume of activation in the sensorimotor cortices in sham and head injured rats in response to 5% CO_2_ challenge as shown in 2b. The mean is the average of 16 brain areas listed in 2c. The red boxes are the % mean of differences. The dashed horizontal lines are 95% confidence intervals. Note that only the temporal cortex for head injury (black) is greater than sham (gray) *** *p* < 0.001

When comparing the mean volume of activation (MVA) between sham and head injured rats for the whole brain (171 brain areas) there was no significant (*p* = 0.229) difference in BOLD signal with a mean difference (MD) of 1.351 across all brain areas (Supplementary Data Fig. 1). However, there were region specific differences in sensitivity to hypercapnia. The brain regions with the greatest decrease in MVA were the prefrontal cortex (Fig. 1), somatosensory cortex (Fig. 2) and basal ganglia. The bar graphs in Fig. 1a show the individual mean score (dots) for each brain area making up the prefrontal cortex together with the total mean ± SD. These brain areas are listed in the estimation graph below (Fig. 1c). The difference in MVA was significant (*p* < 0.05) with a mean difference of -8.75 between sham and head injured rats. The estimation graph shows the individual MVA for sham (gray) and head injured (black) rats (left Y axis) and the differences between them (red box, right Y axis). The change in BOLD signal over time is shown in Fig. 1b. A repeated measures 2-way ANOVA showed a significant interaction between time and condition [F_(149, 16390)_ = 2.083, *p* < 0.0001]. However there was no significant difference between conditions [F_(1, 110)_ = 0.09987, *p* = 0.752].The results for the somatosensory cortex show a significant decrease in MVA in response to hypercapnia (*p* < 0.01) with a mean difference in of -17.81 between sham and head injured rats (Fig. 2a). Of the seventeen brain areas comprising the somatosensory cortex only the temporal cortex for head injury (black) is greater than sham (gray) (Fig. 2c). The change in BOLD signal over time is shown in Fig. 2b. A repeated measures 2-way ANOVA showed a significant interaction between time and condition [F_(149, 32780)_ = 2.517, *p* < 0.0001], but again there was no significant difference between conditions [F _(1, 220)_ = 0.02418, *p* = 0.876]. The basal ganglia also showed a significant (*p* < 0.05) decrease in MVA to hypercapnia with a mean difference of -19.25.

**Fig. 3:**
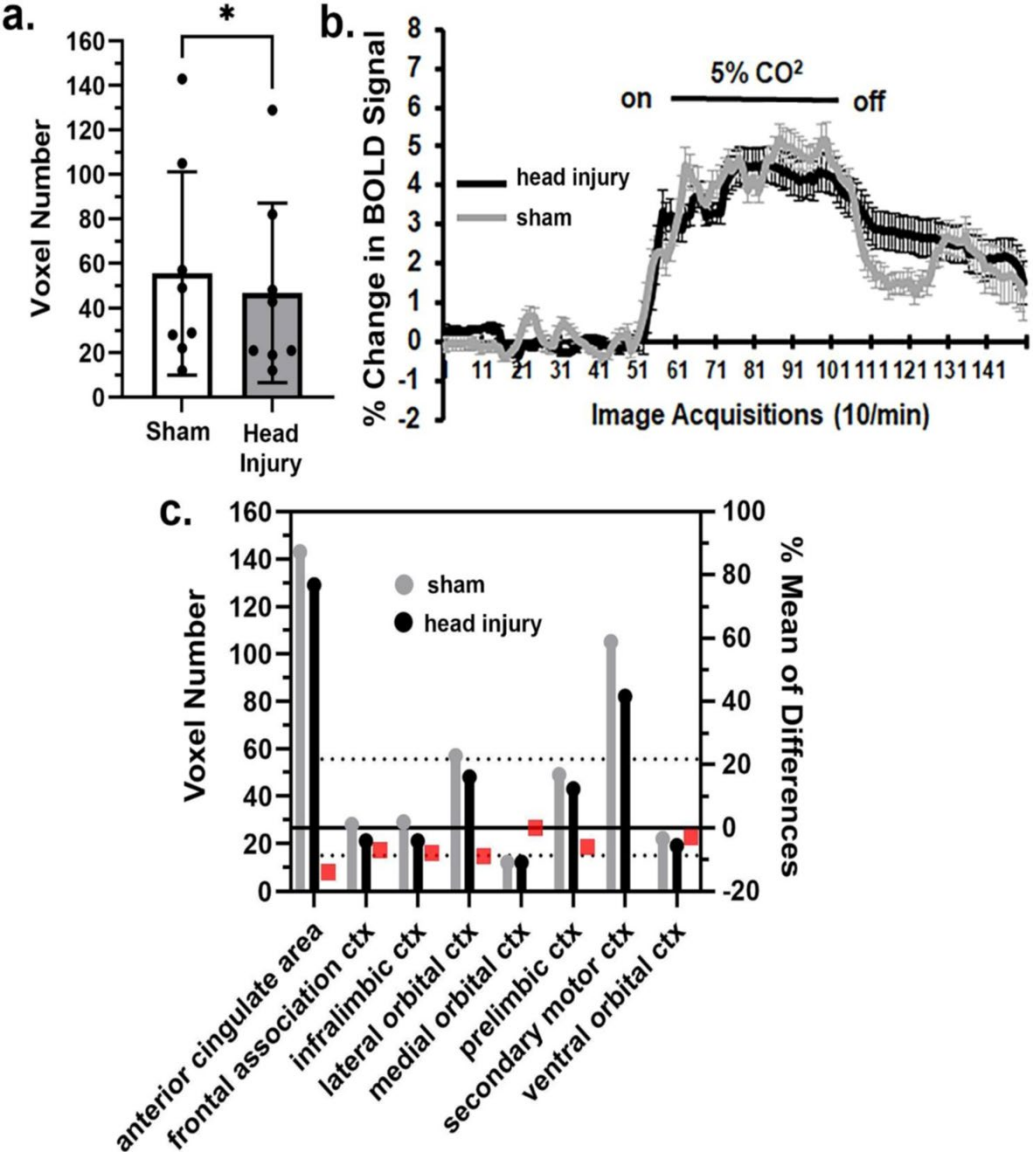
Thalamus response to CO_2_. Shown in 3a are bar graphs (mean ± SD) of the mean volume of activation i.e. number of voxels comprising the thalamus in sham and head injury rats in response to 5% CO_2_ challenge as shown in 3b. The mean is the average of 26 brain areas that comprise the thalamus. The difference in the mean volume of activation for each of these individual areas is shown in the 3c. The red boxes are the % mean of differences. The dashed horizontal lines are 95% confidence intervals. Note that only the reuniens, ventromedial, and zona incerta for head injury (black) are less than sham (gray) ** *p* < 0.01

**Fig. 4:**
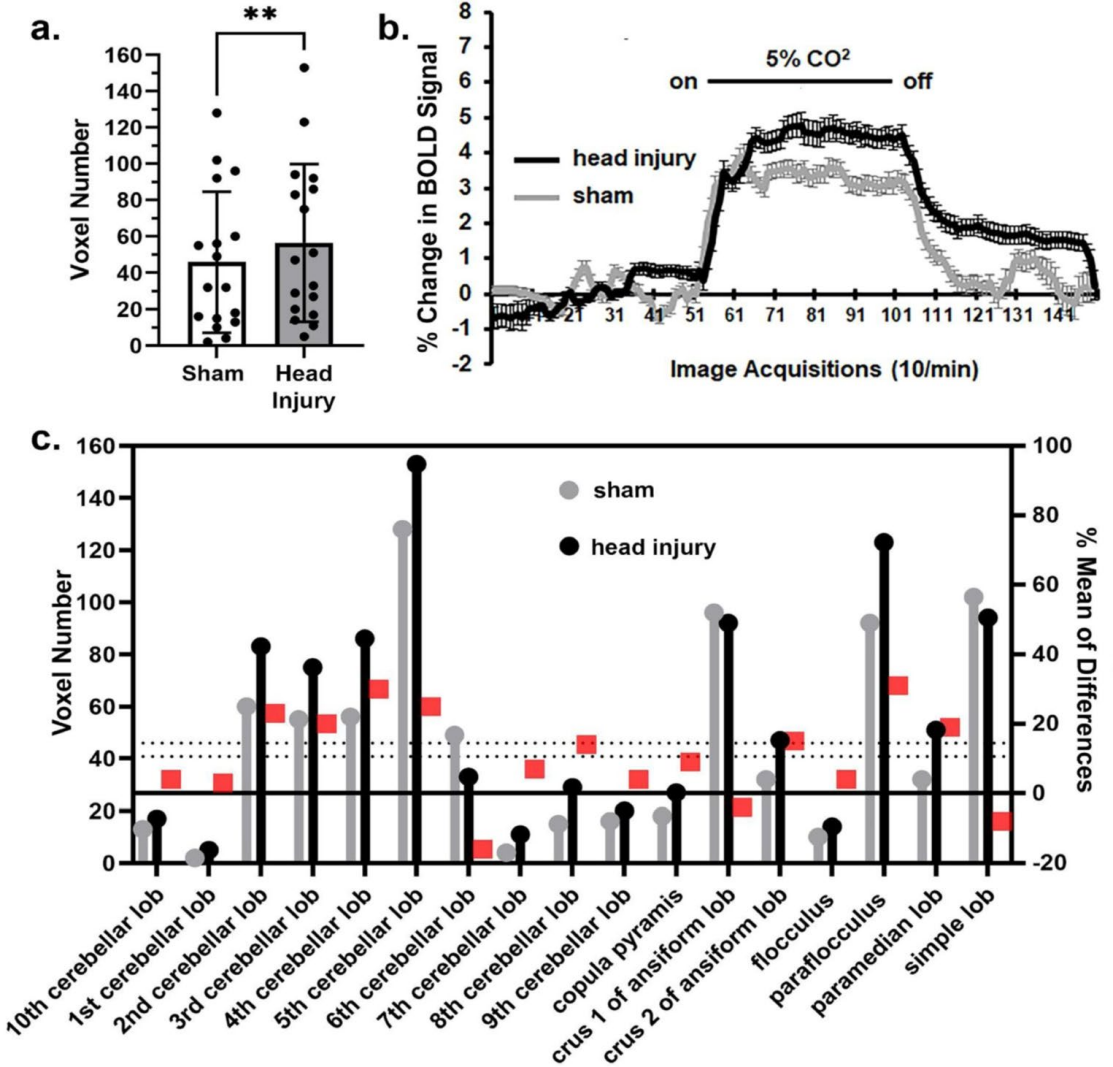
Cerebellum response to CO_2_. Shown in 4a are bar graphs of the mean volume of activation in the cerebellum in sham and head injured rats in response to 5% CO_2_ challenge as shown in 4b. The mean is the average of 17 brain areas shown in the bottom 4c. Note in every case with the exception of the 6th cerebellar lobule, crus 1, and simple lobule the values in head injured rats (black) are greater than the sham (gray). ** *p* < 0.01

There were four brain regions, thalamus (Fig. 3), cerebellum (Fig. 4), brainstem and olfactory system that showed a significant increase in BOLD signal to hypercapnia. The bar graphs in Fig. 3a show the individual mean score (dots) for each brain area making up the thalamus together with the total mean ± SD. These brain areas are listed in the estimation graph below (Fig. 3c). The difference in MVA was significant (*p* < 0.01) with a mean difference of 6.57 between sham and head injured rats. Note, only the reuniens, ventromedial, and zona incerta for head injury (black) are less than sham (gray) In Fig. 3b there was a significant interaction between time and condition [F_(149, 45594)_ = 5.300, *p* < 0.0001] and a significant difference between conditions [F_(1, 306)_ = 10.95, *p* = 0.001]. The bar graphs in Fig. 4a show the individual mean score (dots) for each brain area making up the cerebellum together with the total mean ± SD. These 17 brain areas are listed in the estimation graph below (Fig. 4c). The difference in MVA was significant (*p* < 0.01) with a mean difference of 10.59 between sham and head injured rats. Note in every case with the exception of the 6th cerebellar lobule, crus 1, and simple lobule the values in head injured rats (black) are greater than the sham (gray). In Fig. 4b there was a significant interaction between time and condition [F_(149, 35164)_ = 7.244, *p* < 0.0001] and a significant difference between conditions [F_(1, 236)_ = 12.49, *p* = 0.0005]. The brainstem areas also showed a significant (*p* < 0.001) increase in MVA to hypercapnia with a mean difference of 6.955, together with the olfactory system (*p* < 0.01) with a mean difference of 19.10. It should be noted that the hippocampus, hypothalamus and amygdala were not significantly affected by mild repetitive head injury as compared to sham controls. The midbrain trended toward enhanced sensitivity to CO_2_ challenge (*p* = 0.0519) with a mean difference of 1.57.

## Discussion

In earlier studies we used hypercapnic challenge to demonstrate the difference in CVR, BOLD signal, and cerebral blood flow (CBF) between anesthetized and awake MRI (Brevard et al. 2003; Sicard et al. 2003). As expected, the CO_2_-induced rise in BOLD signal and blood flow were blunted under anesthesia, hence one of the motivations for doing functional imaging in awake animals (Ferris 2022). In a subsequent study we used hypercapnic challenge to explore changes in BOLD and CBF following stroke in rats showing reduced CVR (Sicard et al. 2006). Indeed, CO_2_-induced changes in BOLD and CBF have been used in the clinic to evaluate the health of cerebral vasculature in several neurological disorders including Alzheimer’s and senile dementia (Sur et al. 2020; Glodzik et al. 2013; Yezhuvath et al. 2012), stroke (Pillai and Mikulis 2015), multiple sclerosis (Pelizzari et al. 2020) and traumatic brain injury (Kenney et al. 2016). A recent study by Liu and colleagues evaluated the reliability of hypercapnia driven CVR as a biomarker for cerebrovascular function and found it was suitable across different scanning platforms and imaging sites for use in longitudinal studies and clinical trials (Liu et al. 2021).

There have been numerous studies using BOLD imaging and hypercapnic challenge to evaluate cerebrovascular function following TBI. With moderate to severe TBI there is a significant decrease in CRV in response to CO_2_ challenge (Kenney et al. 2016). Subjects with traumatic cerebral vascular injury following TBI show reduced global CVR compared to healthy controls (Amyot et al. 2018, 2020). Haber and colleagues reported patients with identifiable, localized brain lesions following TBI show a global decrease in CVR that includes unaffected brain areas (Haber et al. 2018). They concluded that vascular dysfunction is a TBI endophenotype in the absence of structural damage long after head injury. Reports of reduced CRV to CO_2_ challenge are also true in preclinical studies of TBI (Wu et al. 2021; Golding et al. 1999). For example, a unilateral lesion in the somatosensory cortex from an open skull impact shows reduced bilateral cortical CVR, preclinical evidence of a global diminished CBF response to TBI injury in otherwise normal brain areas (Long et al. 2015). In this study using three mild head impacts without evidence of skull damage or contusion we identified three brain regions that showed a decrease in CVR to 5% hypercapnia. The forebrain regions of the prefrontal cortex, somatosensory cortex and underlying basal ganglia, all in proximity to the location of head impact, showed decreased BOLD signal as compared to sham controls. Unexpectedly, we also found several brain regions e.g. thalamus, cerebellum, brainstem and olfactory system that showed enhanced BOLD signal to CO_2_ challenge. However, this is not unfounded. Champagne and colleagues reported an increase in CO_2_-induced CRV in subjects with a history of sports related concussions on a background of reduced CBF and cerebral metabolism suggesting enhanced regulation of blood flow to affected areas with reduced metabolism (Champagne et al. 2019, 2021). This increase in CRV following mild head injury was reported earlier in a pilot study by Mutch et al. (Mutch et al. 2016). However, these results showing an increase in CRV are contrary to the findings in female high school soccer players with a history of high magnitude, head accelerated impacts (Svaldi et al. 2020).

There is an increased prevalence of sports related concussions with behavioral and cognitive symptoms but no evidence of neuroradiological skull damage or contusion (McCrory et al. 2013). What constitutes a “mild” head injury? The Glasgow Coma Scale of 13–15 defines head injury as “mild” based on measures of motor behavior, verbal responses and eye opening, with loss of consciousness and short hospitalization (Jennett and Teasdale 1977). With the advent of CT and MRI, physical damage to brain could be confirmed, adjusting the classification to “mild with complication” (Levin et al. 1992). Organized sports and head trauma in the battlefield added another dimension to the characterization of “mild.” The Centers for Disease Control and Prevention, WHO and American Congress of Rehabilitation Medicine for diagnosing mild head injuries include self-reports of transient confusion, disorientation, impaired consciousness, or dysfunction in memory around the time of the injury - importantly there should be no structural damage as determined with CT or MRI (Cassidy et al. 2004; Silverberg et al. 2023). This study in rats used multiple head impacts, separated over time, all of which were “mild” confirmed by the absence of any structural brain damage by MRI (see Supplementary Fig. 1). Our previous publications on repetitive mild head injury met these criteria (Kulkarni et al. 2019; Cai et al. 2021; Leaston et al. 2021).

The change in CVR was heterogenous. The decrease in CVR in forebrain regions near the site of impact is in keeping with much of the literature reporting cerebrovascular dysfunction. The increase in CVR while unexpected would seem to be consistent with much of the sports related concussions as noted above although the present study did not measure cerebral metabolism and basal cerebral blood flow so the hypothesized cerebrovascular adaptation to a compromised brain areas is uncertain. In three previous studies using the mild head injury protocol described here but with isoflurane anesthesia during head impact, we reported the thalamus and cerebellum were uniquely sensitive to one (Kulkarni et al. 2019, 2020), two (Cai et al. 2021) or three (Kulkarni et al. 2019) head impacts separated by 24 h each. With a single head impact there is a significant increase in apparent diffusion coefficient, a surrogate marker of vasogenic edema in these areas (Kulkarni et al. 2020). The edema is short-lived and recovers with 24 h of head impact. A single hit has no long-term consequences on brain structure, function, or behavior (Kulkarni et al. 2019). With two head impacts there is a significant change in the midbrain dopaminergic system characterized by impaired perivascular clearance and robust gliosis and neuroinflammation three weeks post injury (Cai et al. 2021). With three head impacts there are long-term changes in gray matter microarchitecture and functional connectivity in midbrain dopaminergic system and cerebellum that persist for six wks post injury (Kulkarni et al. 2019). Indeed, the midbrain dopaminergic system was sensitive to hypercapnia in this study trending toward a significant increase in BOLD signal. The three-hit procedure is also characterized by localized increases in BBB permeability that spread to more brain regions with each subsequent head impact (Leaston et al. 2021).

### Data interpretation and limitations

The change in BOLD signal over time to CO_2_ challenge in all brain regions led to some unexpected results. The BOLD signal did not return to baseline in the head injured rats within the 5 min period following the cessation of CO_2_. This may be a neurobiological signature of head injury in this model and under these experimental conditions. More perplexing is the one minute “bump” in BOLD signal at the end of the sham group. Four of the seven rats in the sham group showed this increase. When we reviewed our previous publications of hypercapnic challenge in normal and stroked rats with and without anesthesia (Brevard et al. 2003; Sicard et al. 2003, 2006) we noted that all studies had exposure times between 1 and 3 min of 5% CO_2_ and not the five min used in this study. The five min exposure may have behavioral consequences. A review by Améndola and Weary on the behavioral effects of CO_2_ exposure in rats would suggest this change in BOLD signal may be a stress response (Amendola and Weary 2020). Are these rats showing enhanced arousal and increased cerebral blood flow during this window of time?

As a brief communication there were several areas left unattended, not the least of which is the absence of males. The model was designed to reflect the human experience, i.e., mild repetitive head impacts without anesthesia and during the active period of the circadian cycle. As noted above we have reported a single mild head impact in males performed under light isoflurane anesthesia and during the light period of the L-D cycle resolves in a day. Did the anesthesia during impact contribute to the rapid recovery? We did not test for hypercapnic sensitivity after each head impact, confining this pilot study to CO_2_ challenge only after three head impact and then again, only in females.

### Summary

These studies were designed to better reflect the human experience where the occurrence of mild repetitive head injury is common in organized sports, elderly, and service members. There was no neuroradiological evidence of structural brain damage or contusion. Rats were studied during the dark phase of their L-D cycle when they are active eliminating the confound of circadian variation in neurobiology. Mild head impacts and imaging were done in fully awake rats without the confound of anesthesia. Lack of anesthesia allows for inspection of visual signs that may be associated with transient loss of consciousness (e.g., apnea, absence of hindlimb withdrawal reflex, lying motionless). These studies without anesthesia maximize the clinical relevance and animal model fidelity with respect to human head injuries and facilitate accurate assessment of acute neurobehavioral responses post-impact.

## Electronic supplementary material

Below is the link to the electronic supplementary material.

Supplementary Material 1

## Data Availability

All data have been included in the manuscript as Supplementary files. Raw imaging data in different file formats are available on request.
