# Supplementary material for: Changes in cerebral vascular reactivity following mild repetitive head injury in awake rats: modeling the human experience
Source: Exp Brain Res. 2024 Aug 20;242(10):2433–42. doi: 10.1007/s00221-024-06907-7 (PMC11422282; doi:10.1007/s00221-024-06907-7)
Supplement: Supplementary file 1 — Supplementary Material 1 [file 221_2024_6907_MOESM1_ESM.docx]

**Supplementary figures**

**
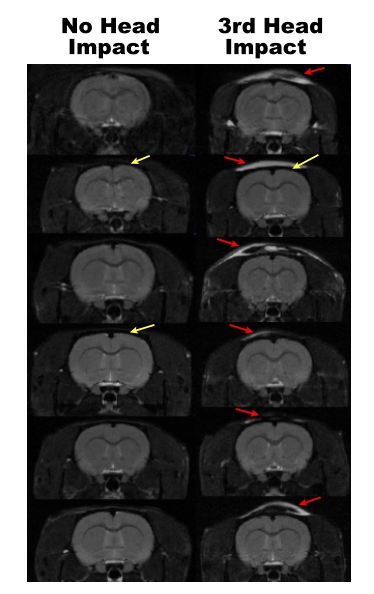
**

**Supplementary Fig. 1** Brain scans following head impact

Shown are MRI images of frontal sections at the level of head impact for no hit and head injured rats. Data were collected at the end of three sham or three head impacts each separated by 24 hrs. The yellow arrows denote the skull (black) overlaying the brain while the white arrows show the edema (white) associated with the skin at the point of head impact. Note the absence of skull damage or any contusion in the underlying gray matter of the cortex.


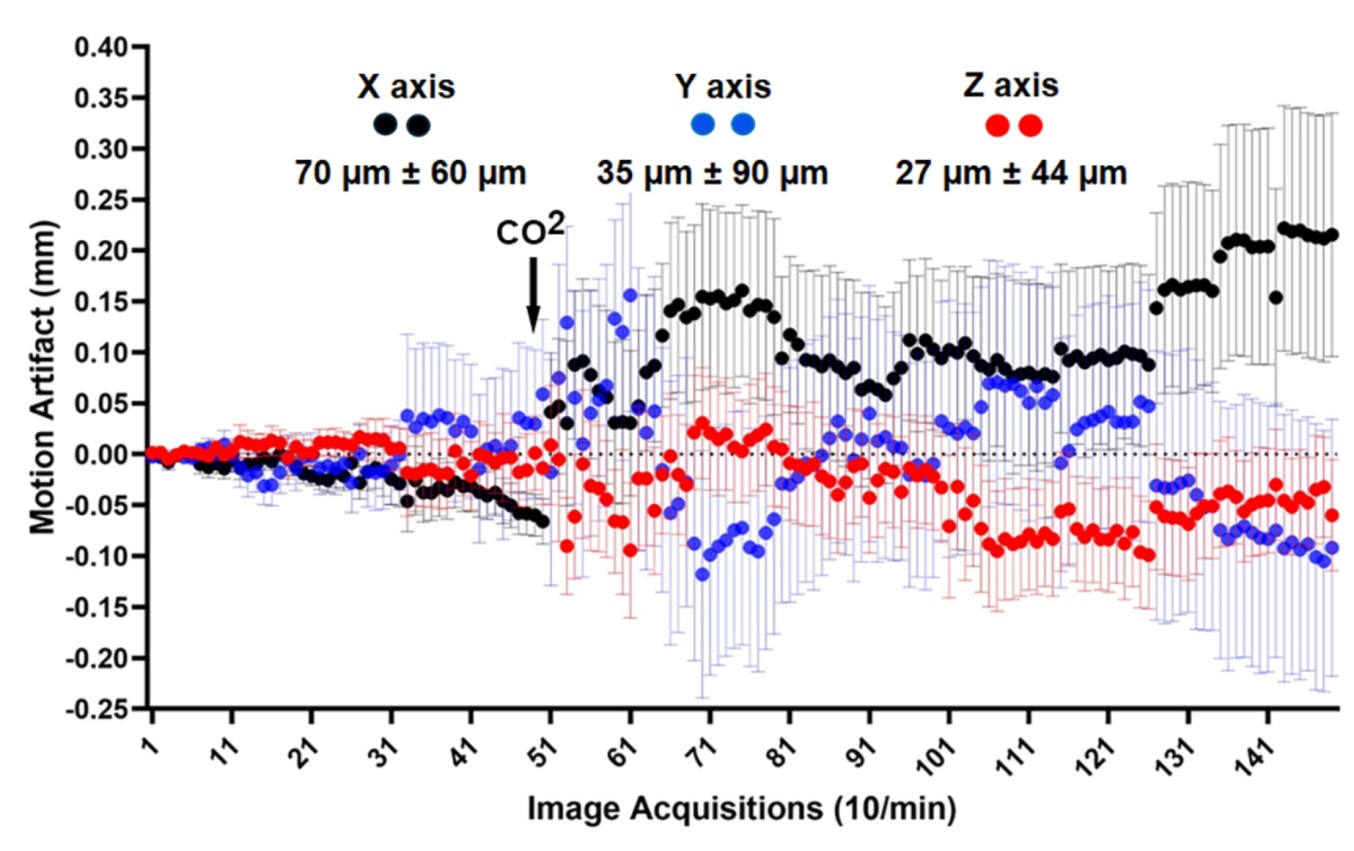


**Supplementary Fig. 2. Motion Artifact**

Shown is a time course of motion artifact over the duration of the scanning protocol. All fourteen of the rats included in the study are combined to show the mean ± SE for the X, Y and Z axis for 150 image acquisitions. The imaging parameters set the in-plane resolution of a pixel at was 312 µm^2^. The average pixel size for the 14 rats ranged between 70 − 27 µm.


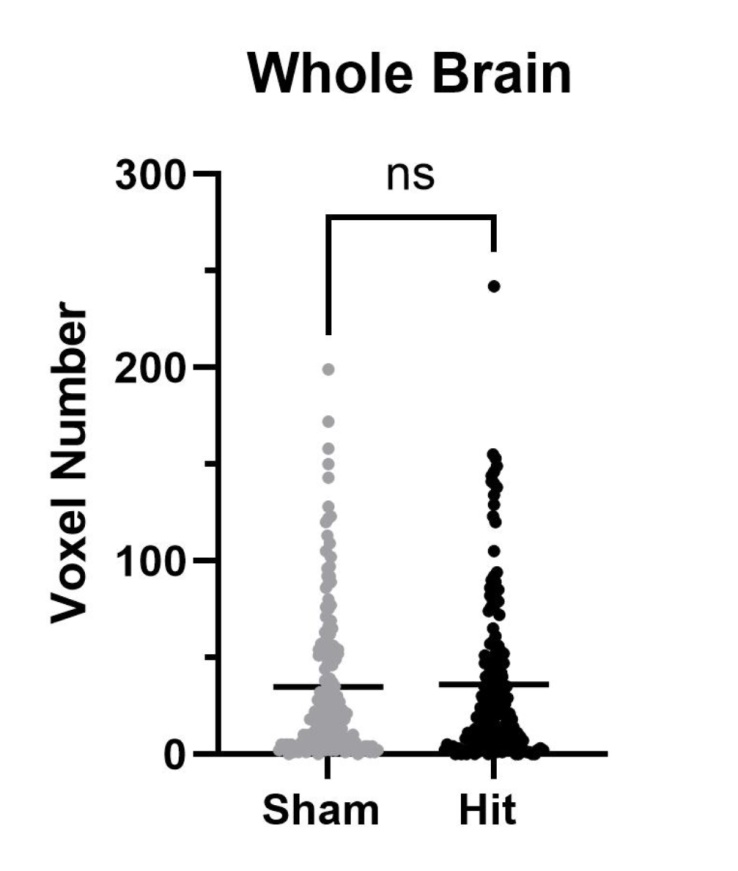


**Supplementary Fig. 3. Whole Brain Cerebral Vascular Reactivity to CO2 Challenge**

Shown are dot plots and the mean (horizontal line) for all 171 brain areas for sham and head injured groups. There was not significant difference.
